# Anticancer Effects of Sacha Inchi (*Plukenetia volubilis*) Shell Extract on Colon Cancer Cells: Integrated GC-MS, LC-MS, Transcriptomic, and Proteomic Analyses

**DOI:** 10.3390/ijms27010234

**Published:** 2025-12-25

**Authors:** Supawadee Osotprasit, Saowaros Suwansa-Ard, Scott F. Cummins, Tianfang Wang, Stuart J. Smith, Tepparit Samrit, Athit Chaiwichien, Narin Changklungmoa, Pornanan Kueakhai

**Affiliations:** 1 Faculty of Allied Health Sciences, Burapha University, Chonburi 20131, Thailand; 63810104@go.buu.ac.th (S.O.); tepparit.sa@go.buu.ac.th (T.S.); 63810103@go.buu.ac.th (A.C.); narinchang@go.buu.ac.th (N.C.); 2 Centre for Bioinnovation, University of the Sunshine Coast, Maroochydore, QLD 4558, Australia; ssuwansa@usc.edu.au (S.S.-A.); scummins@usc.edu.au (S.F.C.); twang@usc.edu.au (T.W.); ssmith16@usc.edu.au (S.J.S.); 3 School of Science, Technology and Engineering, University of the Sunshine Coast, Sippy Downs, QLD 4558, Australia; 4 Food Bioactive Compounds Research Unit, Faculty of Allied Health Sciences, Burapha University, Chonburi 20131, Thailand

**Keywords:** colon cancer, plant extract, sacha inchi, anti-cancer activity, omics

## Abstract

With an aging population and the increase in life expectancy, colorectal cancer is becoming increasingly common. Currently, the mainstay of treatment is chemotherapy, which can cause a variety of side effects. However, herbalists in many cultures have long used herbs to promote health and treat various diseases, including colon cancer. This study investigated the chemical components of shell extracts from *Plukenetia volubilis* (sacha inchi) and their effects on colon cancer cells. First, cytotoxicity against both normal and cancer cells was assessed, followed by cell migration and invasion. Finally, transcriptomic and proteomic analyses were performed to investigate molecular mechanisms. Phytochemical analysis showed a total phenolic content of 134.61 ± 0.27 mgGAE/g extract and a total flavonoid content of 5.75 ± 0.01 mgQE/g extract; moreover, lidocaine (10.57%) and linolenic acid (10.39%) were identified as the most abundant compounds. In vitro, the extract inhibited cell migration, invasion, and colony formation and was associated with potential modulations of the Hippo pathways and epithelial–mesenchymal transition. Therefore, it can be concluded that these extracts are effective in inhibiting the progression of colon cancer cells, and thus, they can prospectively be developed as a dietary supplement or therapeutic agent for the treatment of colon cancer in the future.

## 1. Introduction

The colon is the most common site for malignant tumor origin, and these tumors are associated with very high mortality rates worldwide. In fact, cancers of the colon constitute one of the top three causes of death from cancer; for example, there were over 20 million new cases and 9.7 million deaths due to cancer in the year 2022 alone [1,2]. For this reason, significant effort has been channeled into studying the mechanism of metastasis and developing novel therapeutic approaches.

Tumor metastasis is a complex multistep cascade in which epithelial cells are released from the site of the primary tumor into the blood circulation. This process essentially involves the breaching of the basal membrane by tumor cells, following which they cross the extracellular matrix (ECM) and intravasate into the blood vessels to establish distant metastases [3]. The colonizing cells at new sites must adapt themselves to the new microenvironment and change from a migratory phenotype to a metastatic phenotype [4]. An essential feature of the invasion process is adhesion of tumor cells to the basement membrane, causing the contraction of endothelial cells and protease secretion by the tumor cells, including both Matrix metalloproteinases (MMPs) and serine proteinases, to initiate ECM degradation [5,6]. The endothelial cells also play a role by secreting MMPs and degrading type IV collagen and other matrix proteins in the basement membrane [7]. Therefore, MMPs are considered important biomarkers of invasion, and their inhibition is expected to impede invasion and metastasis.

The strategies for the treatment of colon cancer depend on the tumor location and include surgery, radiation, and targeted chemotherapy. Chemotherapy remains the major therapeutic modality, although it is plagued by a high recurrence rate or non-response to treatment, ranging from 20% to 80% of cases. While chemotherapeutic agents are designed to target malignant cells selectively, normal cells are inevitably affected by their cytotoxic effects, leading to significant side effects. These toxicities increase the risk of mortality, particularly in weakened patients. Therefore, the development of colon cancer treatments with more favorable safety profiles and reduced or minimised side effects is urgently needed. An increasing number of studies show that medicinal herbs contain naturally occurring bioactive compounds proven to be effective against colon cancer and other malignancies, with the added possible advantage of fewer or no side effects compared to conventional therapies.

Sacha inchi (*Plukenetia volubilis Linneo*) is a woody vine of the family Euphorbiaceae, native to the Peruvian Amazon, its distinctive seeds have recently been reported as a putative candidate herb for colon cancer treatment [8]. The seeds are highly valued in the cosmetic, pharmaceutical, and food industries, reflected in a global market estimated at USD 92 million in 2020, which is expected to reach USD 125.8 million by 2027, with a 4.7% CAGR [9]. Previous compositional analyses have determined sacha inchi seeds to contain a low total fat content, while being rich in polysaccharides and polyphenolic compounds, including being high in tannins, lignans, flavonoids, and free phenolic acids. Significantly, higher contents of α-tocopherol have been reported in the shells compared to the seeds, along with a balanced ratio of omega-3 and omega-6 fatty acids. Such unsaturated fatty acids and polyphenols have been attributed to antioxidant, antiproliferative, anti-inflammatory, and metabolic-modulating activities, which contribute to the reduction of diseases such as cancer and obesity [10,11]. Despite such work on sacha inchi extracts, research remains in its infancy, with the bioactive potential of seed shells not being appropriately assessed to date, preventing their development into therapeutic or health-promoting products. This study aimed to isolate crude extracts from the seed shells of sacha inchi and to characterize them by mass spectrometry. We also examined the colon cancer-inhibitory activity of those extracts using in vitro bioassays and multi-omics analysis. Therefore, our results will provide evidence of these extracts’ potential to exert an anticancer property, which could valorize abundant shell waste and strengthen the commercial importance of *Plukenetia volubilis* (*P. volubilis*) in Thailand.

## 2. Results

### 2.1. Phytochemical Analysis of Shell Extract

**Figure 1:**
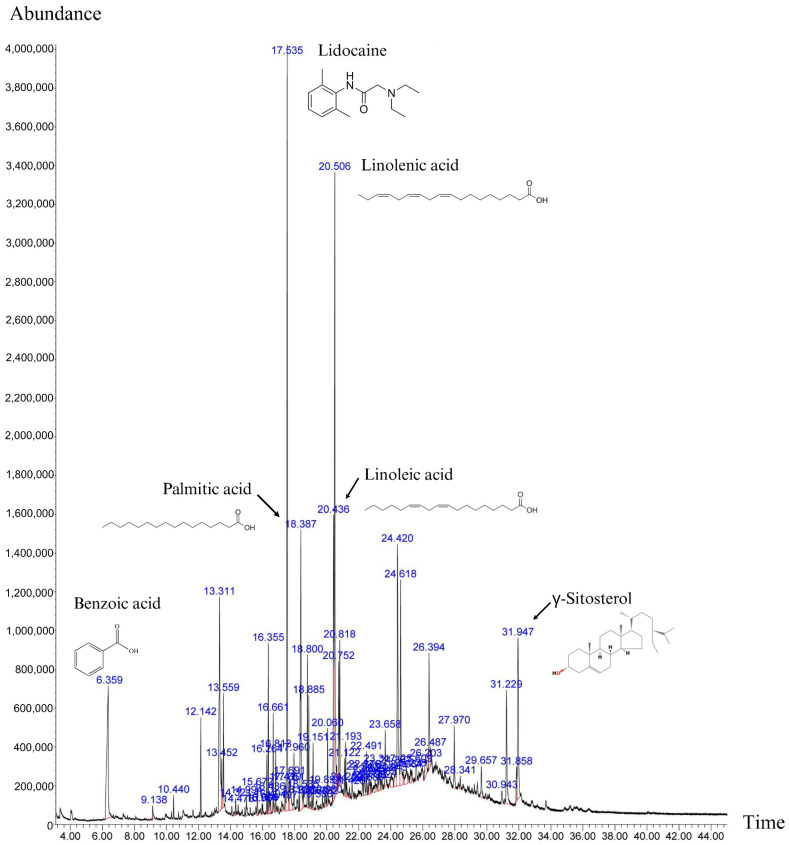
Chromatograph for shell extract gas chromatography–mass spectrometry analysis with high-abundance peaks identified.

After 14 days of continuous extraction in ethanol, 100 g of powdered shell of *P. volubilis* yielded a mass yield (*w*/*w*) of 1.03%. Phytochemical screening of the shell extract presented a total phenolic content of 134.61 ± 0.27 mgGAE/g extract and a total flavonoid content of 5.75 ± 0.01 mgQE/g extract. The Gas chromatography–mass spectrometry (GC-MS) analysis showed 39 distinct peaks (Figure 1), identifying 39 compounds in total. The most abundant compound was identified as lidocaine, followed by linolenic acid, palmitic acid, benzoic acid, linoleic acid, γ-sitosterol, and di-n-octyl phthalate, at 10.57%, 10.39%, 5.83%, 5.82%, 5.36%, 4.23%, and 3.50%, respectively (Table S1).

**Figure 2:**
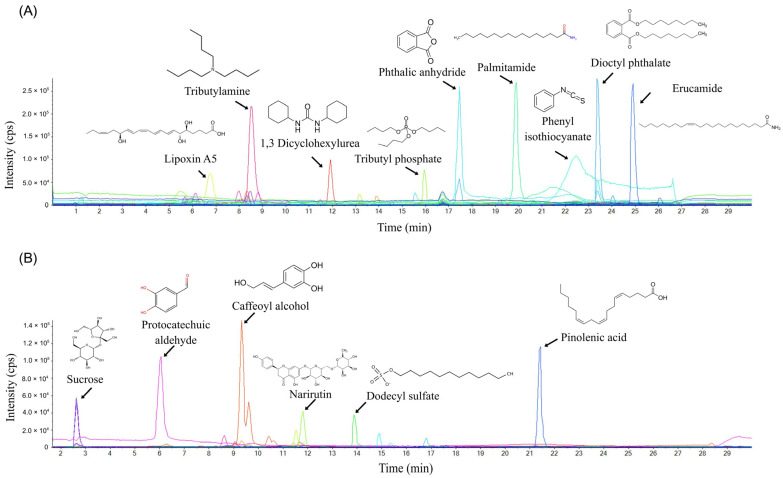
Chromatograph for shell extract Liquid Chromatography–Mass Spectrometry analysis with high-abundance peaks identified. (**A**) positive mode analysis and (**B**) negative mode analysis.

The liquid chromatography–mass spectrometry (LC–MS) analysis identified 57 compounds in positive mode (Table S2) and 23 compounds in negative mode (Table S3) from the shell ethanol extract (Figure 2). The results confirmed the presence of 20 phenolic compounds, including two phenolic acids, in addition to other compounds, such as fatty acids and alkaloids. Many of the compounds identified have previously been shown to exhibit various anticancer properties, including in colorectal cell lines (Table 1).

### 2.2. Cytotoxicity of Shell Extracts Against Colon Cancer Cells

**Figure 3:**
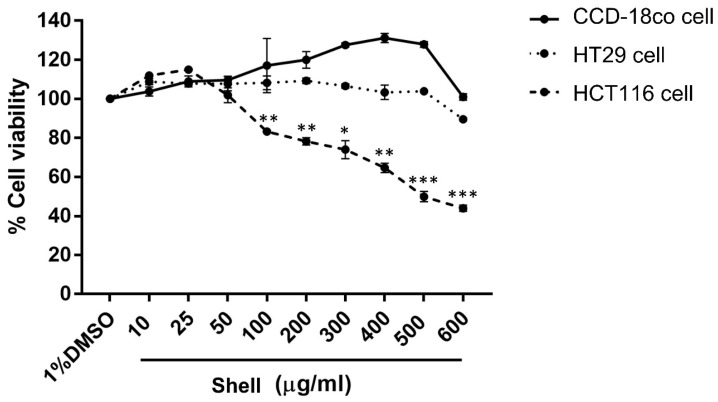
MTT assay results showing cytotoxic effects of the shell extract on normal colon (CCD-18co) and colon cancer (HCT116 and HT29) cell lines. Representative results showed cell viability following treatment with different concentrations of the shell extract. Data are presented as the mean ± SD from three independent experiments (*n* = 3). Statistical analysis was performed using One-way Analysis of Variance (ANOVA) followed by Dunnett’s post hoc test compared to untreated control cells. Asterisks indicate indicate *p*-values: *, *p* < 0.05; **, *p* < 0.01; ***, *p* < 0.001.

MTT assays were performed to evaluate the effect of shell extracts on the cell viability of the normal colon (CCD-18co) and colon cancer (HCT116 and HT29) cell lines at various concentrations (10, 25, 50, 100, 200, 300, 400, 500, and 600 µg/mL) for 24 h. The results indicated no cytotoxicity against CCD-18co and HT29 cells at the tested concentrations, resulting in IC50 values greater than 600 µg/mL. However, HCT116 cells displayed reduced cell viability at all tested concentrations higher than 50 µg/mL. Cell viability was found to be significantly reduced at a concentration of 100 µg/mL, compared to that of 1% Dimethyl Sulfoxide (DMSO) (control), with the IC50 determined at 527.77 µg/mL. These results indicated that the shell extract became increasingly cytotoxic and inhibited cell growth at higher concentrations against HCT116 cells while remaining nontoxic to normal colon cells (Figure 3). To confirm that the observed effects in functional assays were not due to cytotoxicity, only concentrations that kept cell viability within the 95–105% range were chosen for further migration, invasion, and colony formation experiments.

### 2.3. Shell Extract Inhibited the Migration and Invasion of Colon Cancer Cells

**Figure 4:**
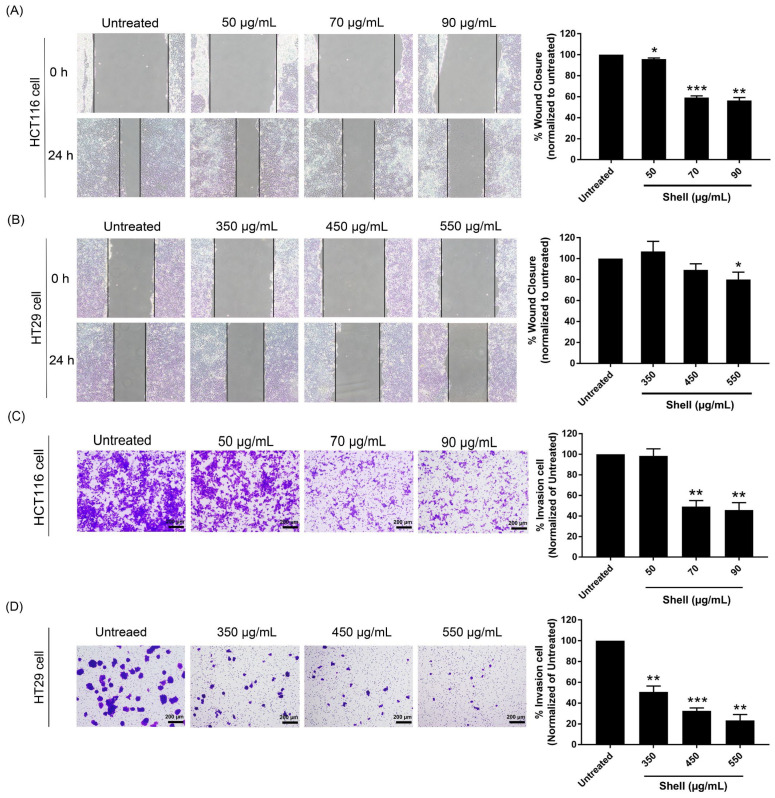
Images of the morphology and quantification of colon cancer cells treated with shell extract at 0 and 24 h for assessing cell migration and invasion. Shell extract inhibited the migration of (**A**) HCT116 and (**B**) HT29 cells, as shown by the wound closure percentage. Shell extract inhibited the invasion of (**C**) HCT116 and (**D**) HT29 cells, as shown by the comparative invasion percentage. The scale bars in (**C**,**D**) represent 200 µm. Data were presented as the mean ± SD from three independent experiments (*n* = 3). Statistical analysis was performed using One-way Analysis of Variance (ANOVA) followed by Dunnett’s post hoc test compared to untreated control cells. Asterisks indicate *p*-values: *, *p* < 0.05; **, *p* < 0.01; ***, *p* < 0.001.

To investigate whether shell extract affected HCT116 and HT29 cell migration, wound scratch assays were performed at concentrations of 50, 70, and 90 µg/mL for HCT116 cells (Figure 4A) and 350, 450, and 550 µg/mL for HT29 cells (Figure 4B). The results showed that shell extracts at concentrations of 50 and 550 μg/mL for HCT116 and HT29 cells, respectively, significantly decreased the percentage of wound closure at 24 h compared with the untreated group. Furthermore, the results showed wound closure rates of 95.80%, 59.27%, and 56.49% for 50, 70, and 90 μg/mL, respectively, in HCT116 cells. In contrast, HT29 cells achieved wound closures of 106.93%, 89.21%, and 80.11% at 350, 450, and 550 µg/mL, respectively. Cell invasion was assessed using a Transwell assay. Data from this experiment showed that shell extracts applied to HCT116 (Figure 4C) and HT29 (Figure 4D) cells significantly decreased the percentage of invasion at concentrations of 70 and 350 μg/mL, respectively, compared with the untreated group. Therefore, these results indicate that the shell extract inhibited migration and invasion in colon cancer cells (HCT116 and HT29).

### 2.4. Effect of Shell Extract on Colony Formation of Colon Cancer Cells

**Figure 5:**
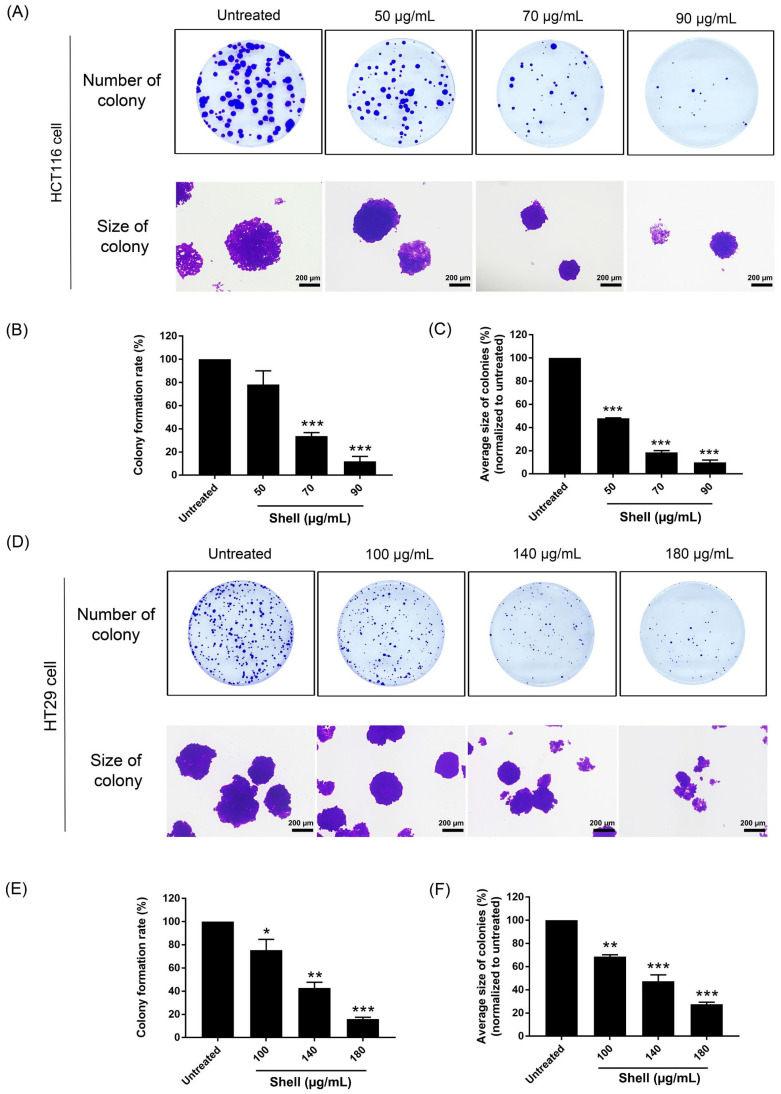
Shell extract inhibited colony formation. (**A**) Number and size of colonies of HCT116 cells treated with shell extract concentrations of 50, 70, and 90 µg/mL (**B**,**C**) show the percentage of colony formation and the average size of HCT116 colonies, respectively. (**D**) Number and size of colonies of HT29 cells treated with shell extract concentrations of 100, 140, and 180 µg/mL (**E**,**F**) show the percentage of colony formation and average size of HCT116 colonies, respectively. The scale bars in (**A**,**D**) represent 200 µm. Data in the histograms were presented as the mean ± SD from three independent experiments (*n* = 3). Statistical analysis was performed using One-way Analysis of Variance (ANOVA) followed by Dunnett’s post hoc test compared to untreated control cells. Asterisks indicate *p*-values: *, *p* < 0.05; **, *p* < 0.01; ***, *p* < 0.001.

A colony formation assay was used to determine the effect of shell extract on the number and size of colon cancer cell colonies using concentrations of 50, 70, and 90 µg/mL for HCT116 cells (Figure 5A–C) and 100, 140, and 180 µg/mL for HT29 cells (Figure 5D–F). The results showed that shell extract significantly inhibited colony formation at 70 µg/mL in HCT116 cells and 100 µg/mL in HT29 cells compared with the untreated group. Therefore, these results indicate that the shell extract inhibited colony forming in colon cancer cells (HCT116 and HT29).

### 2.5. Transcriptomic Analysis of Shell Extract Treatment

We also determined the effect of shell extract treatment on gene expression in HCT116 and HT29 cells, which was profiled via RNAseq analysis. The results of the shell treatment for HCT116 cells revealed a total of 15,605 genes, of which 7846 were upregulated and 7759 were downregulated. For HT29 cells, the results revealed a total of 15,506 genes, of which 7797 were upregulated and 7709 were downregulated compared to those in the untreated group. Of the significantly upregulated differentially expressed genes (DEGs), 73.15% (4646 genes) were shared between the HCT116 and HT29 cell lines, while 66.41% (4412 genes) of the significantly downregulated DEGs were shared. According to the Gene Ontology (GO) analysis, high expression was observed in the extracellular space, extracellular region, and plasma membrane (Figure S1).

**Figure 6:**
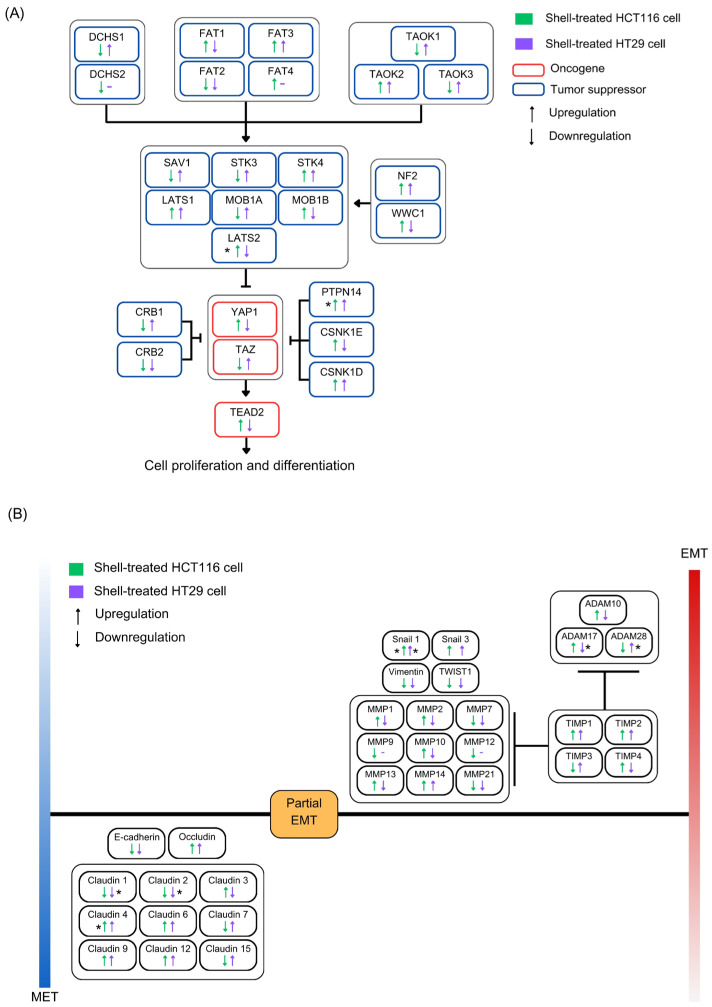
Gene expression involved in the (**A**) upstream Hippo and (**B**) downstream epithelial–mesenchymal transition (EMT) cancer pathways following shell extract treatment of the colon cancer cell lines HCT116 and HT29. The blue and red bars represent the level and intensity of gene expression. EMT and MET are non-binary reversible processes that emphasise the plasticity of cells to transit between these two states. Statistical analysis was performed using a paired *t*-test compared to untreated control cells. Asterisks indicate *p*-values: *, *p* < 0.05.

A detailed analysis of the expression data revealed that pathways related to cell growth, migration, and invasion were identified following the expression of the upstream (Hippo) pathway and the downstream pathways (Epithelial–Mesenchymal Transition (EMT) and Mesenchymal–Epithelial Transition (MET) [20]). The results showed significant upregulation of LATS2 and PTPN14 genes in HCT116 cells compared with the untreated group (*p* < 0.05) (Figure 6A, Figure S2 and Table S4). Furthermore, the results for downstream of EMT and MET gene expression showed that the snail1 and claudin4 genes were upregulated in HCT116 cells; snail1, ADAM28, and claudin1 and claudin2 were upregulated in HT29 cells, while ADAM17 was downregulated, compared with the untreated group (*p* < 0.05) (Figure 6B, Figure S3 and Table S4). Transcriptomic analyses showed that the inhibitory effects of the shell extract on colon cancer cells may be associated with the potential modulations in upstream Hippo signaling and EMT-related pathways, since these pathways were highly enriched with significantly altered genes.

### 2.6. Effect of Shell Extract Treatment on Protein Abundance in Colorectal Cancer Cells

**Figure 7:**
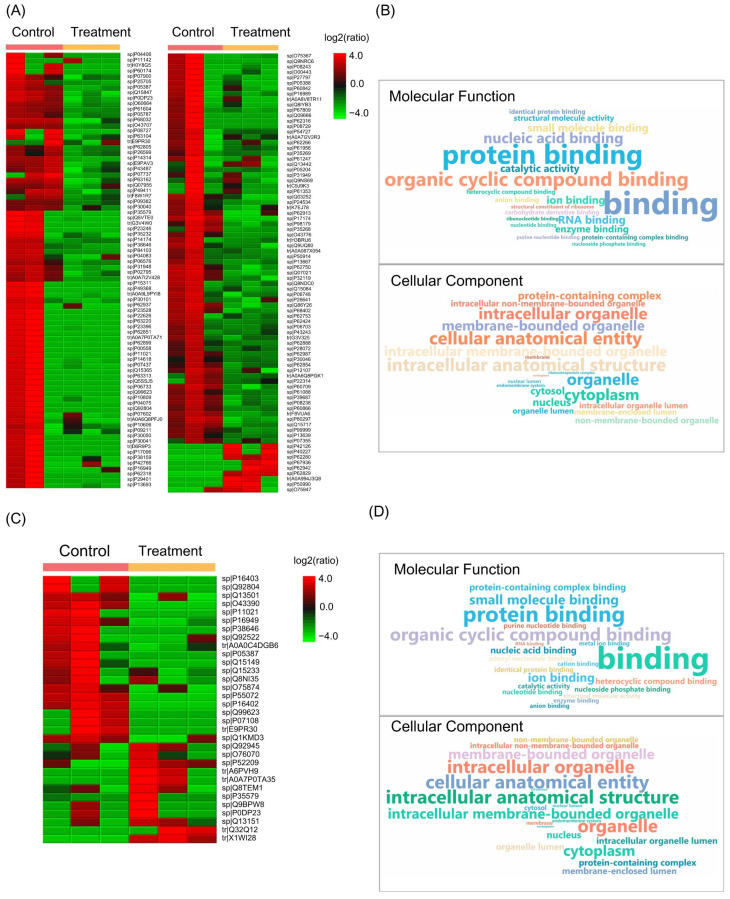
To further elucidate the proteomic changes caused by the treatment with shell extract, untargeted proteomic analysis was performed on HCT116 and HT29 cells after treatment with 100 µg/mL and 450 µg/mL extract, respectively. Heat maps (**A**,**C**) illustrate the relative abundance of differentially expressed proteins in treated HCT116 and HT29 cells compared to their respective controls. Word clouds (**B**,**D**) visualize the Gene Ontology enrichment analysis of the identified proteins, highlighting the most prevalent cellular components and molecular functions associated with the shell extract-induced proteomic changes in HCT116 and HT29 cells, respectively.

Semi-quantitative proteomic analysis revealed a distinct effect of shell extract treatment on the protein expression profiles of HCT116 and HT29 cell lines. Overall, 161 and 32 proteins were found to be differentially abundant in HCT116 and HT29 cells following shell extract treatment, respectively, when compared to untreated controls (Figure 7A,C). For both cell types, the majority of identified proteins exhibited lower abundance upon shell extract treatment and were assigned to similar molecular functions and cellular components (Figure 7B,D). The GO enrichment analysis of standard molecular functions included various binding activities such as protein binding and organic cyclic compound binding. However, nucleic acid binding was more significantly enriched in HCT116 cells, whereas small molecule binding was more dominant in HT29 cells. In relation to cellular localization, both cell types were enriched for proteins located at intracellular and membrane-bounded organelles (Tables S5 and S6).

**Figure 8:**
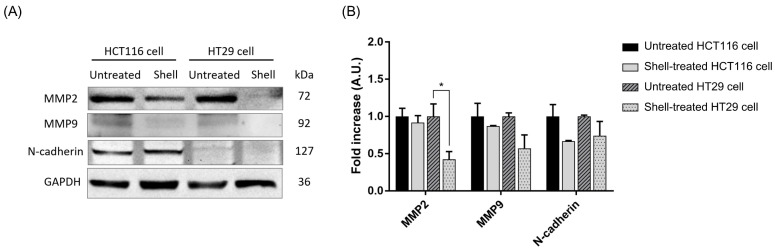
The expression of key proteins associated with the EMT pathway following treatment with shell extract was investigated by Western blotting. Representative immunoblot images showing the protein expression levels of MMP2, MMP9, N-cadherin, and GAPDH (as a loading control) in both untreated and shell-treated cells are provided (**A**). Protein band intensities were quantitated and normalized against GAPDH to demonstrate the influence of shell extract on the abundance of these target proteins (**B**). Data in the histograms were presented as the mean ± SD from three independent experiments (*n* = 3). Statistical analysis was performed using a paired *t*-test compared to untreated control cells. Asterisks indicate *p*-values: *, *p* < 0.05.

Western blot analysis showed that proteins responded differently in the two cell lines. Expression levels of the MMP2 protein were considerably lowered in HT29 cells after treatment with shell extract, but in HCT116 cells, no marked alteration was recorded. For MMP9 and N-cadherin, the statistical analysis showed no significant difference between either cell line or the untreated control group (Figure 8). Collectively, although modulation of these markers was not uniformly robust, the observed reduction in MMP2 in HT29 cells closely aligns with the previously described inhibited migratory and invasive phenotype.

## 3. Discussion

This study demonstrates that sacha inchi shell extract inhibits colon cancer cell migration, invasion, and proliferation, primarily through modulation of the Hippo and EMT pathways, with lidocaine and linolenic acid as key bioactive compounds. Unlike prior research focused on the nutritional value of sacha inchi shells [30], our findings highlight their medicinal potential, particularly in targeting molecular mechanisms of colon cancer progression.

Most current research into sacha inchi focuses on nutritional value of the seeds. Until recently, the shell extract had been reported to contain total phenolic compounds (TPCs) ranging from 9.5 to 15.3 mg GAE/g depending on the extraction solvent used, a 10-fold reduction compared to the TPC identified in this study [30]. The most common solvents previously utilized were organic mixtures such as acetone–water–acetic acid (80:19:1) and methanol–acetone–water–acetic acid (40:40:10:1) at 60 °C [30]. Another study, specifically examining shell extracts, found TPC levels of 3.24 and 5.04 mg GAE/g dry weight in the shells when 20% (*v*/*v*) aqueous ethanol was used at 70 °C for 15 min [31]. However, a process in which the shell was immersed in 50% aqueous ethanol for 24 h and then heated at 70 °C for 2 h yielded a much higher TPC value (129.9 mg GAE/g) compared to pure ethanol or water [32]. Other extraction methods, such as microwave and hot water extraction, have also been reported, yielding TPCs of 41.97 mg GAE/g [33] and 74.8 mg GAE/g dry weight [15], respectively [34]. Interestingly, overnight shell heating at 50 °C had a minor effect on TPC levels [34]. Our extracts produced higher TPC levels, potentially due to the extended soaking and incubation process. This extra time may have helped break down the plant’s cell walls, making it easier to extract phenolic compounds compared to using alternative solvents or higher temperatures, while preventing thermal degradation [35]. In earlier studies, phenolics from shell extracts were identified as flavonoids (such as naringenin, hesperidin, and kaempferol) and phenolic acids (including p-coumaric acid, 4-hydroxybenzoic acid, gallic acid, sinapic acid, and caffeic acid) [31]. Alternatively, within our research group [36], we previously reported that the husk extract contained most of the naringenin and lidocaine. In this study, our findings showed much higher amounts of lidocaine and linolenic acid, which have not been reported previously. While linoleic acid is an omega-6 fatty acid commonly found in plants, lidocaine was considered a synthetic compound derived from existing plant alkaloids, however more recently, lidocaine has been identified as a naturally occurring compound in multiple species of Dioscorea, a genus of tuber commonly cultivated in Thailand and throughout the world [37,38,39]. Additionally, this investigation found both greater structural diversity and abundance of phthalates or phthalic acid esters. These are common plasticiser agents, with industrial variants typically synthesized as derivatives of phthalic anhydride, however, recent studies have demonstrated multiple phthalate compounds exist as secondary metabolites of plants, animals and microorganisms [40,41,42]. These organism-derived phthalates have been shown to exhibit distinct structures from common industrial variants, indicating natural biosynthesis [40]. Additionally, these naturally derived phthalate compounds have demonstrated potent bioactivity as cytotoxic agents against a variety of cancer cell lines [40,41,42]. However, while naturally derived variants exist, there is an abundance of evidence that indicates industrial containment variants of phthalates are readily absorbed by plants and are accumulated alongside other secondary metabolites in oils, saps and potentially shells [43,44,45]. Microplastic prevalence and ineffective waste management has been widely documented in the regions surrounding sacha inchi cultivation sites, illustrating the potential for an elevated prevalence of industrial byproducts, such as phthalates, in the environment [46,47,48,49]. Based on the extraction and analytical procedures of this study, it can be surmised that the composition of the extractions is likely to be a true representation of the composition of the sacha inchi shells utilized, whether all the compounds identified are biosynthetic or sequestration is currently unknown.

The sampling for our previous husk investigation was sourced from the same harvest of sacha inchi as the shells in this study, thereby representing different parts of the same plants. The differences in chemical composition between these two studies and the previous works on the sacha inchi seeds may be due to a variety of factors, including variations in cultivation location or the extraction methodologies and/or in the analytical techniques utilised, such as GC-MS and acetonitrile-based HPLC-MS rather than acidic methanol-based HPLC-MS or compositional differences in the seed compared to the shell and husk.

In our study, we aimed to evaluate the potential of this sacha inchi shell extract to inhibit the growth, migration, and invasion of colon cancer cells. In vitro cell viability assays indicated that the effective cytotoxicity concentrations (70 and 450 µg/mL) for the cancer cell lines (HCT116 and HT29) were nontoxic in normal colon cells. Still, the results showed inhibitory effects of these concentrations on cancer cell migration, invasion, and colony formation, which were consistent with previous studies, demonstrating that lidocaine can inhibit proliferation, invasion, and migration of certain cancer cells [50]. However, differences in response levels across cell lines may reflect the diversity of intracellular mechanisms that respond to the extract’s active components. The results of this study are consistent with earlier research reporting that lidocaine, a volatile phenolic amide, has an antiproliferative effect on SW480 and HCT116 colon cancer cell lines by inhibiting epidermal growth factor receptor (EGFR) through induction of microRNA-520a-3p, which directly targets EGFR [51]. It was also found that lidocaine can inhibit the growth of HCT116 and RKO cells by upregulating apoptosis proteins, including caspase-8, p53, survivin, HSP27, and HSP60 [52]. However, another study reported that while lidocaine can cause cell cycle arrest in HT29 and SW480 cells, it does not significantly affect cell death or proliferation [53]. This finding agreed with that of the current study, which found the extract more specifically bioactive towards HCT116 cells; these showed significantly higher sensitivity when compared to normal colon cells and HT29 cells. Such differential sensitivity could be due to differences in their biological characteristics. Although HCT116 cells are derived from a carcinoma at a more advanced stage than HT29 cells (Duke’s D) [54,55], their poorly differentiated and fast-growing nature makes them more susceptible to cytotoxic agents than the slower, well-differentiated HT29 cells (Duke′s C) [54,55]. These results indicate the potential of the extract to be further developed into targeted anti-cancer agents.

Our transcriptome assay results indicated that the shell extract may be associated with the potential modulations in upstream Hippo pathway, which could affect cell proliferation, survival, and translation in colon cancer cells, a critical signaling cascade that regulates cell growth and apoptosis. In particular, compared to the untreated group, the extract increased the expression of the tumor suppressor genes LATS2 and PTPN14 in HCT116 cells and decreased the expression of the oncogenes YAP1 and TEAD2 in HT29 cells, suggesting targeted modulation of cancer cell growth. These results are consistent with a previous study on lidocaine, a major constituent in the shell extract. This compound has gained attention in cancer research due to its anti-cancer activity in several cell lines, inhibition of cell growth, invasion, and motility, and induction of apoptosis [56,57]. Moreover, this was consistent with previous research showing that the husk extract, composed mainly of naringenin and lidocaine, can inhibit migration and invasion via the EGFR pathway [36]. For instance, lidocaine’s suppression of the PI3K/Akt pathway, which indirectly influences Hippo signaling by reducing MST1/2 inhibition, may explain our observed gene expression changes [58]. This connection highlights a potential mechanism by which the extract exerts its effects, bridging our molecular findings to established signaling pathways. In contrast, the Hippo mechanism, a key pathway in regulating cell proliferation and apoptosis, plays a role in cancer inhibition by activating LATS1/2, which, in turn, inhibits YAP/TAZ entry into the nucleus and induces the expression of tumor-growth-related genes [59]. However, at present, there is no direct evidence that lidocaine can significantly affect the phosphorylation of MST1/2 or LATS1/2 or the translocation of YAP/TAZ. However, lidocaine may act indirectly through the inhibition of PI3K/Akt, as it was found that this pathway can also inhibit MST1/2 [58]. Since lidocaine can inhibit the PI3K/Akt pathway in several cancer models, such as liver cancer, lung cancer, and neuroblastoma, this leads to the assumption that it may affect the Hippo pathway through an indirect mechanism, as it can inhibit MST1/2, which reduces the phosphorylation of LATS1/2 and causes more activation of YAP [58]. Additionally, linolenic acid is an omega-3 fatty acid with anti-cancer effects, and in hepatocellular carcinoma, its levels are lower than usual. Linolenic acid has been shown to increase FXR expression, which, in turn, inhibits the Wnt/β-catenin pathway, thereby reducing cancer cell growth [59]. Furthermore, this acid has been shown to reduce cell proliferation, migration, and invasion by inhibiting FASN and inducing apoptotic pathways in colon cancer [60]. Interestingly, ω-3 PUFAs, such as DHA and EPA, were found to induce the phosphorylation of YAP and its retention in the cytoplasm through the activation of MST1/2–LATS1 via GPR40/120 and PKA, resulting in the activation of the Hippo pathway, which reduced growth and induced apoptosis in colon cancer cells [61]. This suggests that linolenic acid, which is in the same group as ω-3 PUFAs, may have the potential to induce the Hippo pathway in the same way. Thus, both lidocaine and linolenic acid have been shown to exhibit anti-cancer activity through mechanisms that may be linked to the Hippo pathway. It has also been found that sacha inchi oil contains a high amount of Omega-3 fatty acids [62]. Lidocaine may exert its anti-cancer effects indirectly by inhibiting PI3K/Akt, which, in turn, affects Hippo signaling, while linolenic acid has been shown to maintain YAP levels in the cytoplasm and directly inhibit cancer cell growth through the Hippo pathway.

Transcriptomic and proteomic analyses revealed that the shell extract inhibits cancer cell metastasis via molecular regulatory mechanisms, particularly by inhibiting the EMT process and regulating the balance between MMPs and tissue inhibitors of metalloproteinases (TIMPs), which are important factors in cancer cell invasion and metastasis [61,63]. After treatment with the shell extract, the potential modulation of downstream targets of EMT was significantly downregulated, indicating that this signaling pathway may be associated with this process, which is critical for cancer cell invasion [64]. Moreover, proteomic data revealed that most of the altered proteins, which exhibited protein-binding properties, were directly involved in the EMT process, further supporting the extract’s anti-metastatic potential [64]. Additionally, the extract had direct inhibitory effects on enzymes involved in the degradation of the ECM, especially the downregulation of MMP9 and MMP2 and the increase in TIMP-1 and TIMP-2 in cancer cells, which promotes a negative balance of ECM destruction [65,66], reflecting a dual regulation mechanism between MMPs and TIMPs [66,67]. It is well known that MMP2 and MMP9 are important enzymes that are often highly expressed in aggressive tumors and degrade the ECM, especially the basal membrane layer. These enzymes facilitate cancer cell infiltration and metastasis [65,66], while TIMPs act as regulators of MMP activity and play a role in preventing excessive ECM destruction [66,68]. Therefore, the maintenance of a balance between MMPs and TIMPs is considered an important mechanism that plays a role in slowing down cancer cell metastasis [68]. In these results, while transcriptomic and proteomic data initially indicated a potential suppression of EMT by the shell extract, closer examination of individual markers reveals a far more complex and nuanced interplay. Specifically, the observed upregulation of Snail1, a canonical EMT-inducing transcription factor, appears paradoxical to a straightforward EMT suppression model. This paradox underlines the multi-targeted nature of the bioactive components of the shell extract, which can have conflicting or context-dependent effects on different regulatory nodes in the EMT network. It is likely that, while the extract impairs some downstream effectors or parallel pathways associated with the mesenchymal phenotype, it simultaneously induces upstream regulators, such as Snail1, through independent mechanisms; alternatively, this could be a compensatory cellular response or an off-target effect. Therefore, rather than a simple, unidirectional block of EMT, these results point toward a complex dysregulation of the EMT program. Additional functional experiments and expression analyses of other key EMT markers, such as E-cadherin and Vimentin, as well as nuclear localization of Snail1, are needed to detail the net influence of the shell extract on EMT dynamics in these colon cancer cells.

Based on gene expression, the results of this study provide clear empirical evidence that sacha inchi shell extract can exert an anti-migration and anti-invasion effect on colon cancer cells through upregulating the levels of proteins involved in invasion and cancer cell transformation, especially MMP proteins, namely MMP2 and MMP9; these have an important role in the degradation of the ECM, which is an initial step in cancer cell metastasis [69,70]. Although the decrease in MMP2, MMP9, and N-cadherin, which are important markers of EMT, was not statistically significantly different from that in the untreated group, this trend indicates the potential of the extract to inhibit EMT, which is an important mechanism by which cancer cells can lose their epithelial cell characteristics and transform into a more motile mesenchymal cell type [70]. This is consistent with the trend toward invasiveness inhibition and shows that the changes at the protein level were more pronounced than those at the gene level, reflecting post-transcriptional regulation, which may be the primary mechanism of the shell extract’s inhibition [69]. Moreover, gastric cancer cells downregulate N-cadherin and vimentin expression when exposed to lidocaine [71], which is associated with downregulation of MMPs and N-cadherin, and another study showed that husk extract can downregulate MMP-2 and MMP-9 in cancer cell lines [36], similar to the results of this study.

This study demonstrates that the shell extract has the potential for an anticancer effect; however, the limitation of the present study is that it relies solely on in vitro cell culture models. The Hippo and EMT/MET pathways have emerged from our data as important mediators of the observed effects, but these molecular mechanisms need further confirmation. In this regard, the authors of this article concede this limitation and propose to perform comprehensive follow-up experiments with specific pathway inhibitors, siRNA knockdown, and rescue assays to establish definitive cause–effect relationships. Furthermore, specific cell death mechanisms were not characterized in this phase to strictly isolate migration inhibition from cytotoxicity artifacts; these will be addressed in the subsequent investigations. Again, to bridge the gap between findings at the benchtop and possible clinical applications, there is a need for in vivo studies using appropriate animal models. Subsequent efforts will need to be made in studying antitumor efficacy, safety profile, and pharmacokinetics of the extract in vivo before consideration of the active principles can be applied in medicinal product development.

## 4. Materials and Methods

### 4.1. Plant Materials and Extraction

**Figure 9:**
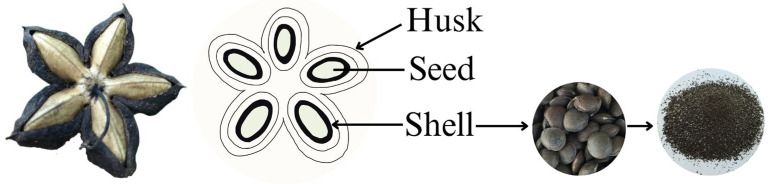
Anatomical structure of sacha inchi fruit, highlighting the shell.

Sacha inchi (*P. volubilis*) inner seed shells (Figure 9) were air-dried and obtained from TAI.C.M.S. standard industrial Co., Ltd. (Chiang Rai, Thailand). A maceration extraction with 95% ethanol was carried out for 14 days. The extract was filtered through a thin white cloth, and the residue was reextracted with 95% ethanol until the supernatant became clear. The combined supernatant was centrifuged at 12,000× *g* for 10 min at 25 °C and filtered through a 6 μm Whatman^®^ qualitative filter paper (Grade 3, Merck, Darmstadt, Germany). The filtrate was evaporated using a rotary evaporator at 56 °C, and the crude extract was dissolved in 100% DMSO to prepare a stock solution at 1 mg/mL, which was stored at −20 °C.

### 4.2. The Total Phenolic Content (TPC)

TPC of the shell extracts was determined using the Folin–Ciocalteu spectrophotometric assay, according to procedures reported by [72,73]. A calibration curve was generated using gallic acid as a standard phenolic compound over the concentration range 4.7–300 μg/mL. In summary, 20 µL of the stock solution of the shell extract was added to 100 µL of 10% (*w*/*v*) Folin–Ciocalteu reagent in a 96-well plate. The mixture was left to incubate in the dark at room temperature for 5 min, after which the mixture was neutralized with 80 µL of 25% (*w*/*v*) sodium carbonate solution. After a further 20-min incubation, the absorbance of the reaction mixture was measured at 760 nm using a microplate spectrophotometer (VersaMax microplate reader, Marshall Scientific, Hampton, VA, USA) against distilled water as a blank. Total phenolic content was expressed as micrograms of GAE per milligram of dry extract. All experiments were performed in triplicate.

### 4.3. The Total Content of Flavonoids (TFC)

The TFC of the shell extracts was determined by the aluminum chloride colorimetric assay as per [61]. A calibration curve was prepared using quercetin as the standard flavanol at concentrations ranging from 4.7 to 300 μg/mL. In short, 20 µL of the shell extract stock solution was mixed with 180 µL of 2% (*w*/*v*) aluminum chloride solution in a 96-well plate. After a 10-min incubation at room temperature, the absorbance was read at 415 nm on a microplate spectrophotometer (VersaMax microplate reader, Marshall Scientific, Hampton, VA, USA) against a distilled water blank. TFC was expressed as milligrams of quercetin equivalents (QE) per milligram of dry extract. Assays were carried out in triplicate.

### 4.4. Gas Chromatography–Mass Spectrometry Analysis

An aliquot of dried shell extract was diluted in ethanol. The sample was injected into a gas chromatography–mass spectrometry (GC-MS) system consisting of a gas chromatograph (Agilent 7890B, Agilent, Santa Clara, CA, USA) and a mass spectrometer detector (Agilent 5977B, Agilent, CA, USA). A ZB-5MS UI (30m × 0.25 mm, 0.25 µm film thickness, Agilent, CA, USA) capillary column was used with coated material of a 0.25 µm film thickness. The inlet was set to 280 °C and split mode at a 10:1 ratio. Helium was used as the carrier gas, adjusted to a column velocity of 1 mL/min. The temperature program was as follows: the initial temperature was 80 °C, which was increased to 100 °C at 5 °C/min, then to 300 °C at 8 °C/min, and finally held at 300 °C for 16 min; the total run time was 45 min. Components were identified by comparing retention times and peak *m*/*z* ratios to those in the National Institute of Standards and Technology (NIST) database.

### 4.5. Liquid Chromatography–Mass Spectrometry

The phytochemical composition of the shell extracts was investigated using an UltiMate™ NCS-3500RS (Thermo Fisher Scientific, Waltham, MA, USA) Binary Rapid Separation Nano/Capillary Pumps system coupled with a TripleTOF 6600 mass spectrometer (Sciex, Framingham, MA, USA).

Chromatographic separations were carried out with a mobile phase composition of solvent A: deionised water with 0.1% formic acid, and solvent B: acetonitrile with 0.1% formic acid. The gradient elution program was carried out at a constant flow of 2.5 mL/min at room temperature, at a maximum pressure of 620 bar. The gradient elution profile was as follows: 5% solvent B for 1 min, linearly increased to 95% solvent B over 20 min, maintained at 95% solvent B for 4 min, and returned to 5% solvent B for 5 min.

Mass spectrometric analysis was carried out in both positive and negative ion modes. The total runtime was 30 min, comprising 545 cycles of 3.3029 sec each. Mass spectra were acquired over the ranges 100–2000 Da for MS1 and 50–2000 Da for MS2. For MS1 acquisition, the nebulizer (GS1), drying gas (GS2), and curtain gas (CUR) flow rates were maintained at 50, 60, and 30 psi, respectively, using nitrogen as the nebulizer/drying gas. The ion source temperature was kept at 500 °C. An ion spray voltage of 5500 V was applied in the positive mode, and −4500 V in the negative mode. The declustering potential, collision energy, and collision energy spread were kept at 80 V, 40 V, and 10 V, respectively, for MS2 acquisition.

### 4.6. Cell Culture

The cell lines used were purchased from the American Type Culture Collection (ATCC, Manassas, VA, USA) and included a normal colon cell line (CCD-18co) and two colon adenocarcinoma cell lines (HCT116 and HT29). CCD-18co cells were passaged in Dulbecco’s Modified Eagle Medium (DMEM) supplemented with 1 g/L D-glucose, L-glutamine, 110 mg/L sodium pyruvate, 10% FBS, 10 U/mL penicillin G, and 10 μg/mL streptomycin. HCT116 and HT29 cells were grown in McCoy’s 5A Medium containing L-glutamine, 10% heat-inactivated FBS, 10 U/mL penicillin G, and 10 μg/mL streptomycin. All cell lines were kept at 37 °C in a humidified atmosphere containing 5% CO_2_. Cultures were refed 2–3 times per week.

### 4.7. MTT Assay

The viability of CCD-18Co, HCT116, and HT29 cells was evaluated using the MTT colorimetric assay as described previously [36]. All cytotoxicity experiments were conducted in triplicate. Cells were seeded into 96-well plates at a density of 1 × 10^4^ cells per well and allowed to attach for 24 h at 37 °C in a humidified 5% CO_2_ atmosphere. Upon attachment, the culture medium was aspirated, and cells were exposed to various concentrations of shell extract (10, 25, 50, 100, 200, 300, 400, 500, and 600 μg/mL) or to 1% DMSO (negative control) for 24 h. Thereafter, the medium was replaced with 100 μL of fresh medium containing 0.5 mg/mL MTT and further incubated for 2 h at 37 °C. The resultant formazan crystals were then dissolved by adding 100 μL of DMSO to each well, and the plates were gently shaken for 15 min. Absorbance was then read at 570 nm on a microplate spectrophotometer (VersaMax, Marshall Scientific, Hampton, VA, USA) with background subtraction at 690 nm. Cell viability was expressed relative to the negative control, 1% DMSO, as a percentage and was calculated based on the following equation:

(1) $$\%\;Cell\;viability=\left(\frac{{Absorbance}_{\left(sample\right)}}{{Absorbance}_{\left(negative\;control\right)}}\right)\times 100$$

### 4.8. Cell Migration Assessment via Wound Scratch Assay

A scratch-wound assay was used to assess the migratory potential of HCT116 and HT29 cells. Cells were seeded in 6-well plates at a density of 1 × 10^6^ cells/well and allowed to reach confluence over 24 h at 37 °C in a humidified 5% CO_2_ atmosphere. A linear wound was then inflicted on the confluent monolayer using a sterile pipette tip. After washing with phosphate-buffered saline to remove detached cells, the cells were treated with shell extract in culture medium at various concentrations, yielding cell viabilities ranging from 95% to 105%. The untreated cells served as a negative control. Wound closure was monitored by imaging at 0 and 24 h post-wounding using an inverted microscope (Olympus CKX53, Tokyo, Japan). The area of the wound was determined using ImageJ software (version 6.1). Cell migration was represented as a percentage wound closure calculated using the following formula:

(2) $$Wound\;Closure\%=\left[\frac{A_{t=0}-A_{t=x}}{A_{t=0}}\right]\times 100$$

A*_t_*_=0_ The area of the wound is measured immediately after scratching (*t* = 0 h).
A_*t*=*x*_ The area of the wound is measured χ hours after the scratch is performed.

### 4.9. Cell Invasion Assessment via Transwell Assay

The cell invasion assay was done using a Transwell invasion assay with a Corning Matrigel basement membrane matrix (Corning 356234, Corning, NY, USA). Matrigel was thawed on ice overnight at 4 °C to avoid premature gelling. All the materials that came in contact with Matrigel were pre-cooled. Matrigel was diluted in serum-free medium to a final concentration of 20 μg/mL, and 100 μL was coated onto the upper surface of Transwell inserts (8 μm pore size; Corning 3464, Corning, NY, USA). Plates were then incubated at 37 °C for 1 h to allow the gel to polymerize. The HCT116 and HT29 cells were seeded in the upper chamber at a density of 5 × 10^4^ cells per well in 250 μL of serum-free medium containing the indicated concentrations of shell extract. The lower chamber was filled with 800 μL of medium supplemented with 20% fetal bovine serum as a chemoattractant. Cells were allowed to invade for [time period] at 37 °C under humidified 5% CO_2_ conditions. Cotton swabs were used to remove the non-invasive cells from the upper surface of the membrane. The invaded cells on the lower surface were fixed with absolute methanol for 5 min, then stained with 0.1% crystal violet for 3 min. The excess stain was washed out using PBS. Invaded cells were counted under a light microscope in randomly selected fields. Experiments were performed in triplicate. The % of cell invasion was calculated by applying the formula:

(3) $$\%\;Invasion=\left(\frac{{Number\;of\;cells\;invade}_{\left(sample\right)}}{{Number\;of\;cells\;invade}_{\left(control\right)}}\right)\times 100$$

### 4.10. Colony Formation

A colony formation assay was performed in triplicate to evaluate the long-term proliferative capacity of HCT116 and HT29 cells upon treatment with the shell extract. Log-phase cells were seeded into 6-well plates at a density of 1 × 10^3^ cells per well and allowed to attach for 24 h at 37 °C in a humidified 5% CO_2_ atmosphere. Thereafter, the medium was aspirated, and cells were treated with various concentrations of the extract for 24 h. Following treatment, the medium was replaced with fresh medium and incubated for an additional 10 days to permit colony formation. Macroscopic colonies were then washed twice with PBS, fixed with absolute methanol for 5 min, and stained with 0.1% crystal violet for 3 min. Excess stain was removed by washing with PBS. Colonies that contained more than 50 cells were counted under a light microscope, while colony size was measured using ImageJ software. Colony formation efficiency was determined by using the following formulas:

(4) $$Colony\;formation\;rate\left(\%\right)=\left(\frac{{Number\;of\;colonies}_{\left(sample\right)}}{{Number\;of\;colonies}_{\left(control\right)}}\right)\times 100$$

(5) $$Average\;size\;of\;colonies\left(\%\right)=\left(\frac{{Average\;size\;of\;colonies}_{\left(sample\right)}}{{Average\;size\;of\;colonies}_{\left(control\right)}}\right)\times 100$$

### 4.11. Transcriptomic Analysis

HCT116 and HT29 cells were seeded in 6-well plates at a density of 1 × 10^6^ cells per well and cultured for 24 h at 37 °C in a humidified 5% CO_2_ atmosphere. Then, the cells were treated with shell extract at concentrations of 100 µg/mL (HCT116) and 450 µg/mL (HT29) for 24 h. After treatment, the cells were harvested by scraping and centrifugation at 1000× *g* for 5 min. The cell pellets obtained were washed with PBS and stored at −80 °C until RNA extraction.

Total RNA was isolated using TRIzol™ Reagent according to the manufacturer’s instructions (Thermo Fisher Scientific, Waltham, MA, USA). In brief, cell pellets were homogenized in 0.75 mL of TRIzol™ Reagent, followed by the addition of 0.2 mL chloroform. The mixture was incubated for 2 min, then centrifuged at 12,000× *g* for 15 min at 4 °C. The aqueous phase containing RNA was transferred into a new tube, and RNA was precipitated with 0.5 mL of isopropanol. After a 10-min incubation at 4 °C, the samples were centrifuged at 12,000× *g* for 10 min at 4 °C. The RNA pellet was washed with 1 mL of 75% ethanol, air-dried, and resuspended in 50 µL of RNase-free water containing 0.1 mM EDTA. The isolated RNA was then incubated at 55 °C for 10 min and stored at −80 °C.

RNAseq was outsourced to a commercial service provider, BGI Hong Kong Tech Solution NGS Lab (Tai Po, Hong Kong; Project number: F23A430002074_HOMkcpqR). Starting from total RNA, the library preparation workflow involved fragmentation, mRNA enrichment using oligo [34] magnetic beads and cDNA synthesis. Double-stranded cDNA was purified, enriched by PCR amplification, and sequenced on the BGIseq-500. The sequencing results have been deposited in the NCBI SRA under accession PRJNA1236814. For bioinformatics, KEGG pathway and Gene Ontology (GO) analyses were performed using BGI’s proprietary in-house data mining system, the Dr TOM approach. The reads were aligned to the NCBI Homo sapiens reference genome (Version: GCF_000001405.39_GRCh38.p13). Differential gene expression (up- or down-regulation) was quantified and reported as log2 fold change (log2FC) based on FPKM values.

### 4.12. Sample Preparation and Protein Digestion

The HCT116 and HT29 cells were seeded in 6-well plates at 1 × 10^6^ cells/well in complete McCoy’s 5A medium and incubated for 24 h at 37 °C in a humidified 5% CO_2_ atmosphere. After incubation, the cells were treated with the shell extract for 24 h at 37 °C. Cells were harvested by scraping and centrifugation at 1000× *g* for 5 min at room temperature. The resulting cell pellets were then washed with PBS and lysed in 600 µL of lysis buffer containing 8 M urea, 0.8 M NH_4_HCO_3_ (pH 8.0), and PMSF. Lysates were sonicated on ice for 2 min at 40% amplitude with 10 s on/off cycles and clarified by centrifugation at 12,000× *g* for 15 min at 4 °C. Supernatants were recovered, and protein concentrations were determined using the Pierce BCA protein assay.

For protein digestion, 100 µg of protein was reduced with DTT (5 µL, 100 mM) for 1 h at 37 °C, followed by alkylation with IAA (20 µL, 100 mM) for 1 h at RT in the dark. The samples were then further incubated with DTT (20 µL, 200 mM) for 60 min at RT. The urea concentration was lowered by diluting the samples with 775 µL of Milli-Q water. The samples were then digested overnight with sequencing-grade modified trypsin at 37 °C and 20 rpm. Following digestion, the samples were acidified with 10% FA to a pH < 3. Tryptic peptides were desalted on Sep-Pak C18 cartridges, dried in a SpeedVac, and reconstituted in 50 µL of 0.1% FA for LC-MS analysis.

LC-MS Analysis Peptide samples (15 µL) were injected into an Exion LC liquid chromatography system (AB SCIEX, Marlborough, MA, USA) and separated on an Agilent AdvanceBio Peptide Map column (2.1 × 150 mm, 2.7). A binary solvent system consisting of 0.1% formic acid (solvent A) and acetonitrile/0.1% formic acid (solvent B) was utilized over a 52-min time gradient. The gradient elution started at 100% with solvent A for the first 5 min, followed by a linear decrease to 60% of solvent A over 40 min, and then to 5% of solvent A over 5 min. Re-equilibration of the column was carried out with 100% of solvent A for 2 min. Blank runs were executed at the beginning and end of each sample batch, while wash sequences were inserted between the individual samples. The peptides were analyzed in positive mode with an electrospray ion source at 5500 V, declustering potential (DP) at 100 V, curtain gas flow at 30 psi, nebulizer gas (GS1) at 12, and an interface heater at 450 °C. A full TOF-MS scan mode (1.62 s) over the mass range of 350–1800 *m*/*z* was operated in a mass spectrometer. Product ion MS/MS spectra were acquired for precursor ions with *m*/*z* values between 50 and 1800, exceeding a threshold of 100 counts, and with charge states from +2 to +5.

### 4.13. Protein Identification and Functional Annotation

Protein identification was performed using PEAKS software (version 7.0; BSI, Toronto, ON, Canada) against the *Homo sapiens* reference genome database (UP000005640), containing 82,485 protein sequences. Raw data analysis was performed by combining de novo sequencing, database search, and PTM identification. An FDR of 0.5% was selected and the corresponding score threshold [(−10*log(p)] calculated. The details of the database search parameters in PEAKS were as follows: precursor ion mass tolerance was set at 5 ppm, fragment ion mass tolerance was 0.1 Da, enzyme specificity was trypsin with up to three missed cleavage sites allowed, mono-isotopic mass for both precursor and fragment ions was selected, cysteine carbamidomethylation was fixed, whereas lysine acetylation, asparagine and glutamine deamidation, methionine oxidation, and pyroglutamate formation from glutamic acid and glutamine were used as variable modifications. The mass spectrometry proteomics data have been deposited to the ProteomeXchange Consortium via the PRIDE partner repository with the dataset identifiers PXD062512 and 10.6019/PXD062512.

GO annotation was carried out using the functional annotation module of Omicsbox software version 3.0.30. By default, BLASTp searching (NCBI, https://blast.ncbi.nlm.nih.gov/Blast.cgi?PAGE=Proteins, accessed on 22 December 2025), GO mapping (EMBL’s European Bioinformatics Institute), and subsequent annotation were performed using the non-redundant Nr database with an e-value cut-off of 10–5 and lineage restriction to mammals. To summarize, the top 20 annotated GO terms from each pair-wise comparison dataset were categorized into molecular function and cellular component domains and presented as word clouds in which the word size was proportional to the frequency of sequences associated with each GO term.

### 4.14. Targeted Western Blot Analysis

Colon cancer cells treated with shell extract were harvested and lysed in cold lysis buffer (8 M urea, 0.8 M NH_4_HCO_3_, pH 8) supplemented with protease inhibitors. Protein concentration was measured using the BCA Protein Assay Kit (#23227, ThermoFisher Scientific, Waltham, MA, USA). Cell lysates (30 µg) were separated on SDS-PAGE and transferred to a 0.2 µm nitrocellulose blotting membrane (Bio-Rad Laboratories Inc., Hercules, CA, USA). The membrane was blocked with 4% skimmed milk at room temperature for 1 h and then incubated with primary antibodies overnight at 4 °C: rabbit polyclonal anti-human MMP2 (#4022), MMP9 (#3852) and N-cadherin (#4061) (1:500 dilution; Cell Signaling Technology, Danvers, MA, USA). The membrane was then washed three times with 1× PBST (PBS with 20% Tween), incubated with HRP-conjugated Goat Anti-Rabbit IgG (H+L) secondary antibody for 1 h at room temperature, and washed three times with 1× PBST. Chemiluminescence imaging was performed, and protein bands quantified using Quantity One software (version 6.1, Bio-Rad Laboratories, Inc., Hercules, CA, USA).

### 4.15. Statistical Analysis

Data are presented as the mean ± SD of at least three independent experiments (*n* ≥ 3). All statistical analyses were performed using GraphPad Prism (version 7.04). Comparisons between two groups were done using a Student’s *t*-test. Comparisons involving more than two groups were done using a One-way ANOVA, followed by Dunnett’s post hoc test for multiple comparisons. A *p*-value of less than 0.05 (*p* < 0.05) was considered statistically significant. The statistical tests used for each experiment are described in the figure legends.

## 5. Conclusions

This research demonstrated that sacha inchi shell extracts possess anticancer properties against HCT116 and HT29 colon cancer cells, exhibiting selective bioactivity toward HCT116 cells. Phytochemical profiling revealed high values of total phenolics and flavonoids. In vitro assays, molecular profiling studies, and protein analysis confirmed the anticancer activities of the detected compounds. Therefore, this study suggested that sacha inchi shell extracts may be a promising candidate for a novel targeted therapy against colon cancer, thereby contributing to the economic value of *P. volubilis* in Thailand. Although initial cellular experiments have shown high anticancer activity, further studies are required to ensure consistency in the composition of the extracts for potential use in dietary supplements or therapeutic agents. Their efficacy, safety, and mechanisms of action should be further validated in more specific in vivo studies.

## Figures and Tables

**Table 1:** Bioactive phytocomponents identified in the shell extract via liquid chromatography–mass spectrometry.

Compound	Class	Activity	References
alpha,beta-Trehalose	Disaccharide	A novel anticancer agent is effective in treating tumors by decreasing oxidative stress, autophagy, and increasing apoptosis.	[12]
Vitamin B6	Pyridine	Reduces the number of tumors in the colon, inhibits angiogenesis, reduces oxidative stress and has anti-colorectal cancer effects.	[13]
Trigonelline	Pyridine	Anti-invasive activities against cancer cells (Hepatoma cells).	[14]
Byakangelicin	Coumarin	Inhibits SHP-1/JAK2/STAT3 signaling and thus blocks tumor growth and motility in breast cancer.	[15]
Echinocystic acid 3-glucoside	Triterpenoid glycoside	Inhibited tumor growth, suppressed proliferation and induced the apoptosis of lung carcinoma cells (A549 cells).	[16]
Isoliensinine	Phenyl alkaloid	Exhibits anti-tumor activity against lung adenocarcinoma (LUAD) both in vitro and in vivo.	[17]
Protoporphyrin IX	Porphyrin	Inhibited oncogenic Ras/MEK can modulate the heme biosynthesis pathway and cancer in vitro and in vivo (colon and breast cancer).	[18]
Sclareol + Na	Diterpene	Induce suppression of the growth of HCT116 tumors established as xenografts in immunodeficient SCID mice.	[19]
Tuberostemonine	Alkaloid	Inhibition of both the AKT and ERK pathways is essential for maximizing the improvement of pulmonary fibrosis.	[20]
Piperine	Alkaloid	Inhibits the canonical Dima Wnt signaling pathway and displays anti-cancer effects on colorectal cancer cell lines.	[21]
*Cis*-9-Hexadecenoic acid	Fatty acid	An anti-inflammatory lipid that helps ameliorate metabolic disorders.	[22]
2-Methoxycinnamaldehyde	Phenyl aldehyde	Anti-inflammatory and anti-apoptotic properties.	[23]
Phenylacetaldehyde	Phenyl aldehyde	Induced ROS deregulated the STAT3/IL-6 pathway, and PAA may be a potential agent targeting breast cancer.	[24]
Mono-2-ethylhexyl phthalate	Phenolic	Progression of CRC through AKT-β-catenin signaling in vitro and in vivo models.	[25]
Palmitamide	Fatty acid	Anti-inflammatory, antioxidant, and immune-enhancing effects and malignant tumors.	[26]
Phenyl isothiocyanate	Phenolic	Induced apoptosis in breast cancer overexpressing HER2 in vitro and in vivo.	[27]
Diphenylamine	Phenylamine	Inhibits proliferation, suppresses the androgen receptor, and reduces protein expression in prostate cancer (BET family).	[28]
Bornyl acetate	Ester	Anti-proliferative in cancer cells (cervix, colon, lung and breast cancer).	[29]

## Data Availability

The datasets generated and analyzed during the current study are publicly available. The raw data from whole transcriptome sequencing are deposited under the BioProject accession number PRJNA1236814 in both the NCBI BioProject and SRA databases, accessible at https://www.ncbi.nlm.nih.gov/bioproject/?term=PRJNA1236814 (accessed on 16 March 2025). Raw data from peptide mass spectrometry are accessible under the accession number PXD062512 at https://www.ebi.ac.uk/pride/archive/projects/PXD062512 in the PRIDE database (accessed on 11 November 2025).

## Supplementary Materials

The following supporting information can be downloaded at: https://www.mdpi.com/article/10.3390/ijms27010234/s1.

## Abbreviations

The following abbreviations are used in this manuscript:

DEGs	Differentially expressed genes
DMSO	Dimethyl Sulfoxide
DP	Decluttering potential
DTT	Dithiothreitol
EMT	Epithelial–Mesenchymal Transition
FA	Formic acid
GC-MS	Gas chromatography–mass spectrometry
GO	Gene Ontology
IAA	Alkylating reagent
LC-MS	Liquid Chromatography–Mass Spectrometry
MET	Mesenchymal–Epithelial Transition
MMP	Matrix metalloproteinases
NIST	National Institute of Standards and Technology
PBS	Phosphate-buffered saline
PBST	Phosphate-buffered saline and 20% Tween
RNAseq	RNA sequencing
PMSF	Phenyl-Methyl-Sulfonyl-Fluoride
QTOF	Quadrupole Time-of-Flight
SRA	Sequence Read Archive
SD	Standard deviation
SDS-PAGE	Sodium Dodecyl Sulfate–Polyacrylamide Gel Electrophoresis
TFC	Total flavonoid content
TPC	Total phenolic content
TIMPs	Tissue inhibitors of metalloproteinases
