# Supplementary material for: Anticancer Effects of Sacha Inchi (Plukenetia volubilis) Shell Extract on Colon Cancer Cells: Integrated GC-MS, LC-MS, Transcriptomic, and Proteomic Analyses
Source: Int J Mol Sci. 2025 Dec 25;27(1):234. doi: 10.3390/ijms27010234 (PMC12785288; doi:10.3390/ijms27010234)
Supplement: Supplementary file 1 [file ijms-27-00234-s001.zip › Supplement.pdf]

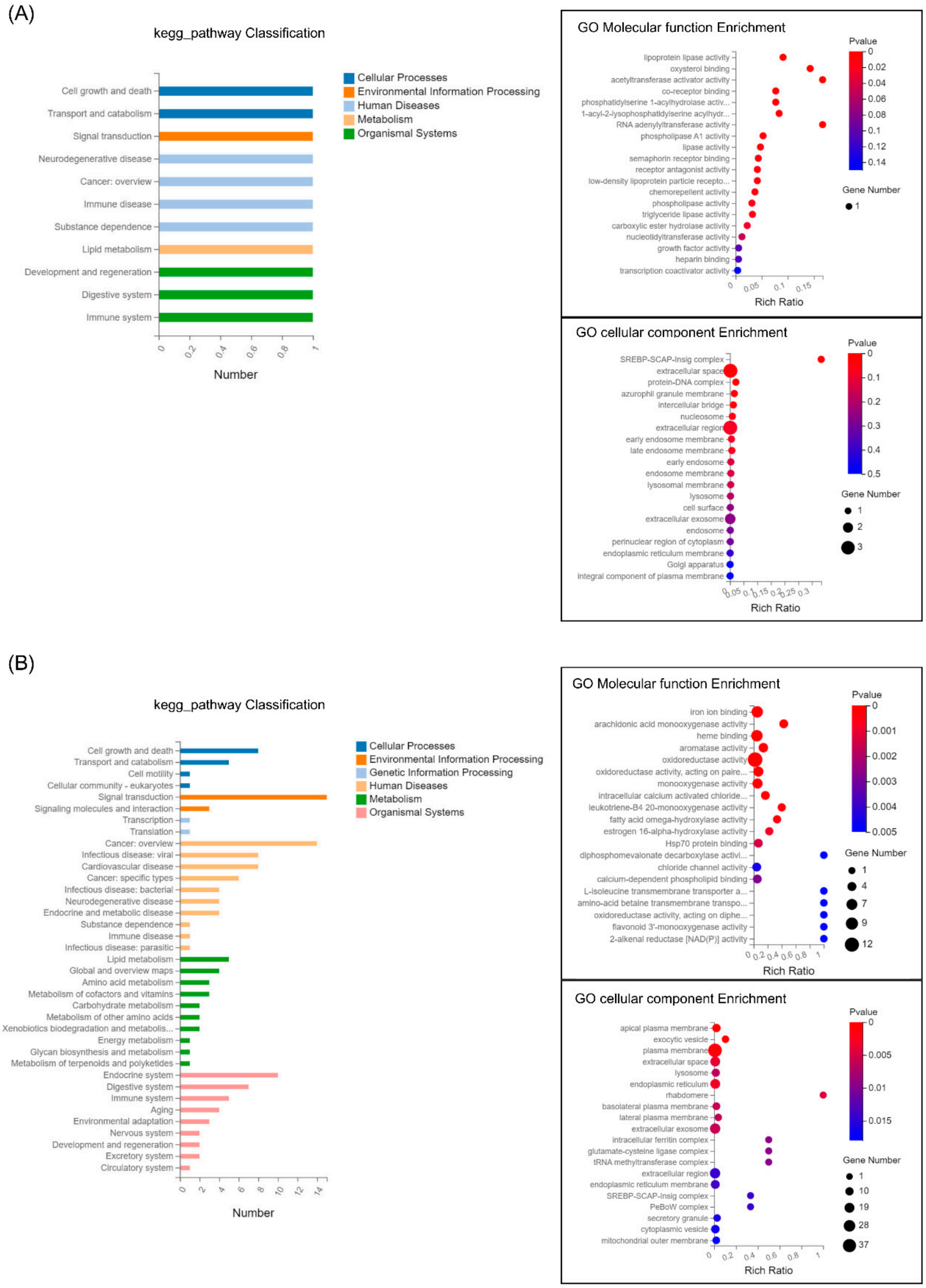

Figure S1. KEGG pathway and GO classification for the differentially expressed genes following shell extract treatment. Classifications for genes in (A) HCT116 cells and (B) HT29 cells. The

KEGG pathway classification provides an enriched string diagram with the corresponding relationship between the target protein and the KEGG pathway. In GO molecular function and GO cellular component enrichment bubble diagrams, the circle size represents the number of enriched genes, and the colour represents the significance of enrichment.

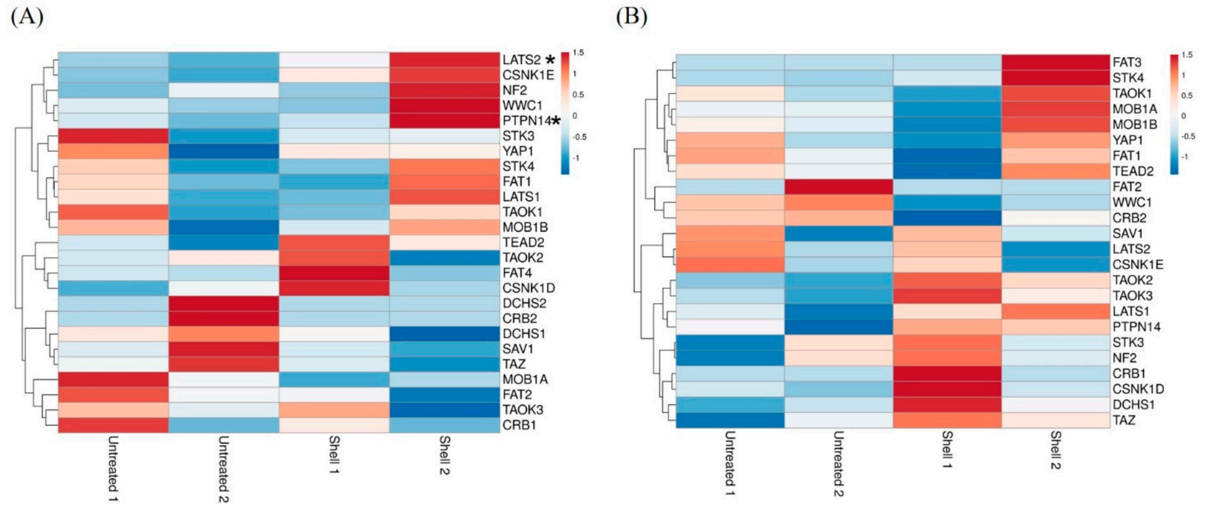

Figure S2. The heatmap for upstream gene (Hippo pathway) expression in HCT116 (A) and HT29 cells (B). Data were compared using a paired t-test, *indicates $p < 0.05$.

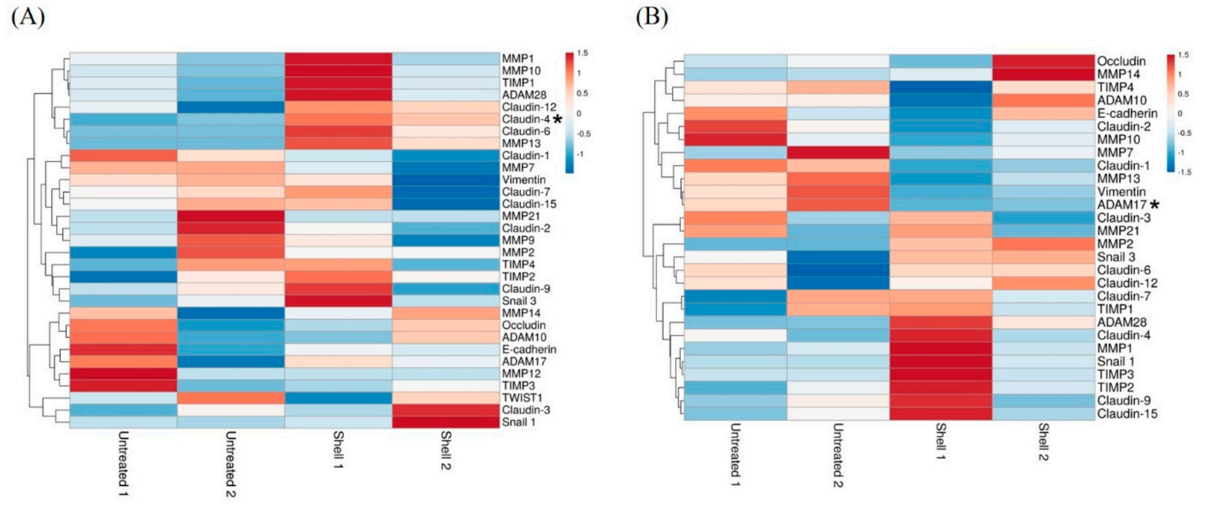

Figure S3. The heatmap for downstream gene (EMT) expression in HCT116 (A) and HT29 cells (B). Data were compared using a paired *t*-test, *indicates $p < 0.05$.
